# Eugene Braunwald: Lições e Histórias do Pai da Cardiologia Moderna

**DOI:** 10.36660/abc.20260349

**Published:** 2026-04-30

**Authors:** Marcus Vinicius Bolivar Malachias

**Affiliations:** 1 Faculdade de Ciências Médicas de Minas Gerais Belo Horizonte MG Brasil Faculdade de Ciências Médicas de Minas Gerais , Belo Horizonte , MG – Brasil; 2 Instituto de Hipertensão Arterial Belo Horizonte MG Brasil Instituto de Hipertensão Arterial , Belo Horizonte , MG – Brasil

**Keywords:** Cardiologia, História, Doenças Cardiovasculares

Em 22 de abril de 2026, recebemos a notícia da morte de Eugene Braunwald, em Boston, aos 96 anos, deixando órfãos milhares de cardiologistas em todo o mundo que, como eu, o consideram consensualmente o pai da cardiologia moderna. Ao receber o honroso convite da Editora dos *Arquivos Brasileiros de Cardiologia* , Glaucia Maria Moraes de Oliveira, para escrever este editorial, deparei-me com o difícil desafio de resumir, em poucas palavras, sua trajetória, seus ensinamentos e sua importância para a nossa especialidade — tarefa que assumo sob a perspectiva pessoal de um cardiologista.

Conheci Braunwald por meio dos livros. Na residência de clínica médica, devorava o tratado de *Medicina Interna de Harrison* , em que Braunwald era o editor principal. Depois, já na residência de cardiologia, nossa bíblia era o seu *Tratado de Doenças Cardiovasculares de Braunwald* .

Desde os primeiros anos da prática médica, assim como ao longo de minha formação, pós-graduação e vivência profissional, sempre me impressionou sua capacidade de liderar pesquisas e produzir conhecimento nas mais diversas áreas da cardiologia: a compreensão do consumo de oxigênio pelo miocárdio, a redução da mortalidade no tratamento da insuficiência cardíaca, a revolução no manejo das síndromes coronarianas agudas, entre tantos outros marcos de sua produção científica, incluindo a clássica e definitiva expressão “tempo é músculo”.

**Figura 1:**
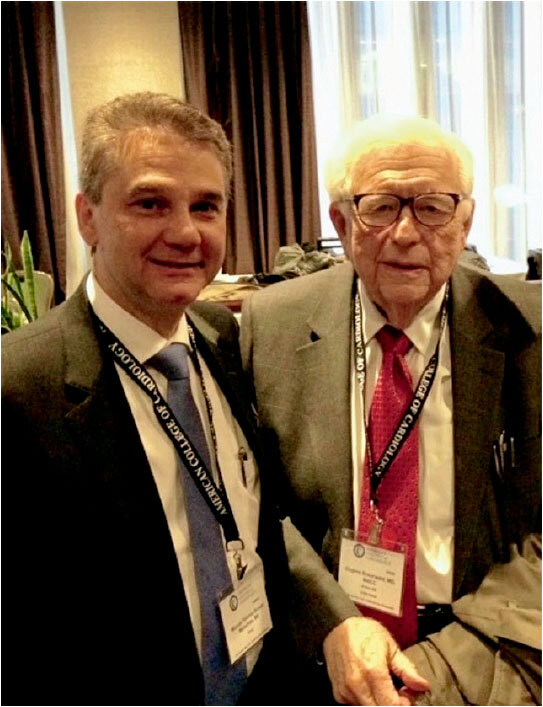
– Marcus Vinicius Bolivar Malachias e Eugene Braunwald, em simpósio do American College of Cardiology, Nova York, NY, EUA, 2016.

Braunwald sempre foi figura central nos grandes congressos médicos. Assisti a várias de suas conferências nas sessões do American College of Cardiology (ACC) ( Figura 1 ), da American Heart Association (AHA) e da European Society of Cardiology (ESC), mas também em suas vindas ao Brasil, nos congressos da Sociedade Brasileira de Cardiologia (SBC).

Lembro-me especialmente de sua conferência no Congresso da ESC de 2013, em Amsterdã, na qual apresentou sua visão dos dez maiores marcos da cardiologia: a criação do eletrocardiograma (Einthoven); o cateterismo cardíaco (Forssmann, Cournand e Richards); a cirurgia cardíaca (Gross e Gibbon); o ecocardiograma (Edler e Hertz); o marca-passo (Zoll) e o desfibrilador externo (Mirowski); a arteriografia coronariana seletiva (Sones); os avanços da cardiologia preventiva (White e o Estudo de Framingham); a criação das unidades coronarianas (Julian); os novos fármacos, como betabloqueadores (Black), inibidores da angiotensina (Ferreira, Cushman e Ondetti) e estatinas (Endo); e a cardiologia invasiva (Gruentzig). ^1^ Naquele dia, compreendi que só é possível atingir o cume científico com plena compreensão dos alicerces do conhecimento.

**Figura 2:**
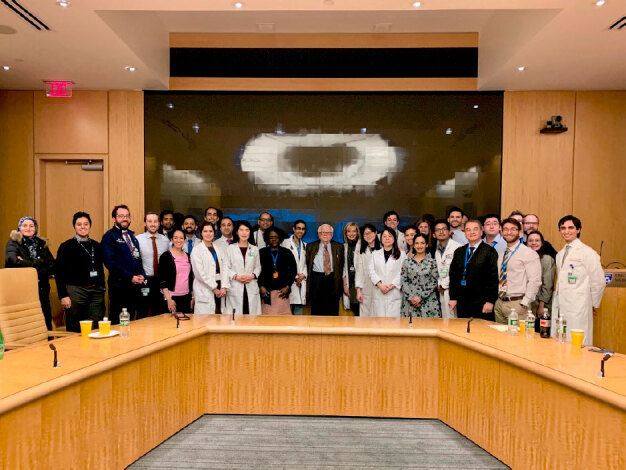
– Eugene Braunwald, em reunião com os fellows do Brigham and Women’s Hospital, Harvard Medical School, Boston, MA, EUA, 2019.

Nos anos de 2019 e 2020, a convite de Peter Libby, realizei pós-doutorado no Brigham and Women’s Hospital (BWH), da Harvard Medical School, sob orientação de Marc Pfeffer e coorientação de Scott Solomon. O primeiro compromisso oficial de todos os *fellows* daquele ano foi uma reunião com Braunwald, no auditório oval do edifício da torre central que leva seu nome, em área nobre do hospital ( Figura 2 ).

Com voz já frágil, porém segura, o professor deu boas-vindas a todos e resumiu sua longa trajetória: da infância em Viena, na Áustria, de onde, jovem judeu, fugiu do regime nazista para a Inglaterra e depois para Nova York, onde se formaria médico; à residência em medicina interna e cardiologia no Johns Hopkins Hospital; ao treinamento no Mount Sinai Hospital; às pesquisas de pós-doutorado no laboratório do Nobel André Cournand, na Columbia University; ao trabalho no National Heart Institute; à chefia do Departamento de Medicina da University of California; até sua chegada a Boston, em 1972, à Harvard Medical School e ao Peter Bent Brigham Hospital (atual BWH), além da criação do TIMI Study Group, em 1984. ^2 , 3^

Braunwald citou Andrew Glenn Morrow como seu “maior mentor” e relembrou que, com ele, publicou em 1959, na revista *Circulation* , a descrição de uma “condição misteriosa”: a cardiomiopatia hipertrófica. ^2^ Ao final da reunião, ressaltou as agruras da trajetória rumo ao êxito na medicina, enalteceu a oportunidade concedida a cada um de nós de treinar em um dos maiores centros mundiais de pesquisa, assistência e ensino e, com autoridade inconteste, cobrou de todos o compromisso com a excelência científica.

Poucos dias depois, participamos da abertura da programação científica anual do BWH, a sessão *Grand Rounds* , coordenada por meu orientador Marc Pfeffer, cujo conferencista era, naturalmente, Eugene Braunwald. O tema era “Diabetocardiologia: uma nova especialidade médica”. Sempre atento às novidades, comentou com entusiasmo os avanços recentes da área.

Como experiente orador, gostava de criar suspense em suas apresentações. Lembro-me do slide com a expressão “ *Surprise!* ”, ao comentar os resultados dos estudos realizados com novos fármacos antidiabéticos, que não apenas demonstravam segurança, mas revelavam, pela primeira vez, redução de desfechos cardiovasculares adversos em pacientes com diabetes tipo 2. ^4 - 7^

Com mais de 1.600 artigos publicados e presença marcante em todos os aspectos da especialidade, Braunwald reescreveu a história da medicina cardiovascular e foi além, triunfando na arte de liderar e integrar diferentes competências: prática clínica, observação médica, ética, pesquisa, registro científico, ensino, difusão do conhecimento, cooperação em equipes eficientes, inovação, desenvolvimento contínuo e formação sistemática de novos profissionais.

Seu legado exemplifica a rara amálgama entre esforço, dedicação, obstinação, observação, método, idealismo e genialidade — combinação que inspira todos aqueles que buscam a excelência.

Mas o fato pessoal mais marcante, e que guardo com carinho na memória, foi o e-mail enviado por ele ao meu orientador — e seu discípulo e amigo — Marc Pfeffer, com cópia para mim, em referência ao meu artigo sobre o valor do NT-proBNP na predição de desfechos em pacientes de alto risco, cujos resultados considerou “bombásticos”. ^8^ Gostava de expressões de efeito. Seu comentário final e assinatura foram inesquecíveis: **“Dynamite! Gene.”**
