# Supplementary material for: Eugene Braunwald: Lições e Histórias do Pai da Cardiologia Moderna
Source: Arq Bras Cardiol. 2026 Apr 30;123(4):e20260349. [Article in Portuguese] doi: 10.36660/abc.20260349 (PMC13398798; doi:10.36660/abc.20260349)
Supplement: Translation

# Translation: Eugene Braunwald: Lessons and Stories from the Father of Modern Cardiology

**DOI:** 10.36660/abc.20260349i

**Authors:** Marcus Vinicius Bolivar Malachias

**Affiliations:** 1 Faculdade de Ciências Médicas de Minas Gerais Belo Horizonte MG Brazil Faculdade de Ciências Médicas de Minas Gerais , Belo Horizonte , MG – Brazil; 2 Instituto de Hipertensão Arterial Belo Horizonte MG Brazil Instituto de Hipertensão Arterial , Belo Horizonte , MG – Brazil

**Keywords:** Cardiology, History, Cardiovascular Diseases

On April 22, 2026, we received the news of the death of Eugene Braunwald in Boston at the age of 96, leaving orphaned thousands of cardiologists around the world who, like me, unanimously regard him as the father of modern cardiology. Upon receiving the honorable invitation from the Editor of the *Arquivos Brasileiros de Cardiologia* , Glaucia Maria Moraes de Oliveira, to write this editorial, I was faced with the difficult challenge of summarizing, in a few words, his trajectory, his teachings, and his importance to our specialty—a task I undertake from the personal perspective of a cardiologist.

I came to know Braunwald through books. During my Internal Medicine residency, I devoured *Harrison’s Principles of Internal Medicine* , in which Braunwald was the lead editor. Later, during my Cardiology residency, our bible was his *Braunwald’s Heart Disease* .

From the earliest years of medical practice, as well as throughout my training, postgraduate education, and professional life, I was always impressed by his ability to lead research and generate knowledge in the most diverse areas of cardiology: understanding myocardial oxygen consumption, reducing mortality in the treatment of heart failure, revolutionizing the management of acute coronary syndromes, among many other milestones of his scientific production, including the classic and definitive expression, “time is muscle.”

Braunwald was always a central figure at the major medical congresses. I attended several of his lectures at meetings of the American College of Cardiology (ACC) ( Figure 1 ), the American Heart Association (AHA), and the European Society of Cardiology (ESC), as well as during his visits to Brazil for the congresses of the Brazilian Society of Cardiology (SBC).

**Figure 1:** – Marcus Vinicius Bolivar Malachias and Eugene Braunwald at a symposium of the American College of Cardiology, New York, NY, USA, 2016.

I especially remember his lecture at the 2013 ESC Congress (1), in Amsterdam, in which he presented his view of the ten greatest milestones in cardiology: the invention of the electrocardiogram (Einthoven); cardiac catheterization (Forssmann, Cournand, and Richards); cardiac surgery (Gross and Gibbon); echocardiography (Edler and Hertz); the pacemaker (Zoll) and external defibrillator (Mirowski); selective coronary arteriography (Sones); advances in preventive cardiology (White and the Framingham Study); the creation of coronary care units (Julian); new drugs—such as beta-blockers (Black), angiotensin inhibitors (Ferreira, Cushman, and Ondetti), and statins (Endo); and invasive cardiology (Gruentzig).^1^On that day, I understood that one can only reach the scientific summit through a full understanding of the foundations of knowledge.

In 2019 and 2020, at the invitation of Peter Libby, I pursued postdoctoral training at Brigham and Women’s Hospital (BWH), Harvard Medical School, under the supervision of Marc Pfeffer and co-supervision of Scott Solomon. The first official commitment of all fellows that year was a meeting with Braunwald in the oval auditorium of the central tower building that bears his name, in a prominent area of the hospital ( Figure 2 ).

**Figure 2:** – Eugene Braunwald in a meeting with fellows from Brigham and Women’s Hospital, Harvard Medical School, Boston, MA, USA, 2019.

With a voice already fragile, yet still firm, the professor welcomed everyone and summarized his long journey: from childhood in Vienna, Austria, from where, as a young Jewish man, he fled the Nazi regime to England and later to New York, where he would become a physician; to residency training in Internal Medicine and Cardiology at Johns Hopkins Hospital; training at Mount Sinai Hospital; postdoctoral research in the laboratory of Nobel laureate André Cournand at Columbia University; work at the National Heart Institute; chairmanship of the Department of Medicine at the University of California; and finally his arrival in Boston in 1972, at Harvard Medical School and Peter Bent Brigham Hospital (now BWH), in addition to founding the TIMI Study Group in 1984.^2 , 3^

Braunwald cited Andrew Glenn Morrow as his “greatest mentor” and recalled that with him he published, in 1959 in *Circulation* , the description of a “mysterious condition”: hypertrophic cardiomyopathy.^2^At the end of the meeting, he emphasized the hardships of the path toward success in medicine, praised the opportunity each of us had been given to train at one of the world’s leading centers of research, care, and teaching, and, with unquestionable authority, demanded from all of us a commitment to scientific excellence.

A few days later, we attended the opening of BWH’s annual scientific program, the *Grand Rounds* , coordinated by my mentor Marc Pfeffer, whose speaker was, naturally, Eugene Braunwald. The topic was “Diabetocardiology: a New Medical Specialty.” Always attentive to innovation, he enthusiastically commented on the most recent advances in the field.

As an experienced speaker, he enjoyed creating suspense in his presentations. I remember a slide displaying the word “Surprise!” as he discussed the results of studies with new antidiabetic drugs, which not only demonstrated safety, but for the first time revealed a reduction in adverse cardiovascular outcomes in patients with type 2 diabetes.^4 - 7^

With more than 1,600 published articles and a remarkable presence in every aspect of the specialty, Braunwald rewrote the history of cardiovascular medicine and went beyond, mastering the art of leading and integrating different competencies: clinical practice, medical observation, ethics, research, scientific reporting, teaching, dissemination of knowledge, cooperation in efficient teams, innovation, continuous development, and the systematic training of new professionals.

His legacy exemplifies the rare amalgam of effort, dedication, perseverance, observation, method, idealism, and genius—a combination that inspires all those who seek excellence.

But the most personal and memorable moment, which I cherish dearly, was the e-mail he sent to my mentor—and his disciple and friend—Marc Pfeffer, copying me, regarding my article on the value of NT-proBNP in predicting outcomes in high-risk patients, whose results he considered “bombastic”.^8^He was fond of emphatic expressions. His final comment and signature were unforgettable: **“Dynamite! Gene.”**
